# Editorial: The notch signaling pathway in lymphoid malignancies

**DOI:** 10.3389/fonc.2023.1216398

**Published:** 2023-05-16

**Authors:** Franco Fais, Dimitar G. Efremov

**Affiliations:** ^1^ Department of Experimental Medicine, University of Genoa, Genoa, Italy; ^2^ Molecular Pathology Unit, IRCCS Ospedale Policlinico San Martino, Genoa, Italy; ^3^ Molecular Hematology Unit, International Center for Genetic Engineering and Biotechnology, Trieste, Italy

**Keywords:** chronic lymphocytic leukemia, T-acute lymphoblastic leukemia, marginal zone lymphoma, signaling, notch

In humans, the NOTCH family includes four members (NOTCH1 through NOTCH4). Its ligands belong to the Delta-like (DLL1, DLL3 and DLL4) and the Jagged (JAG1 and JAG2) families. NOTCH family molecules regulate processes such as proliferation, differentiation and cell death and are involved in the maturation of lymphoid cells, determining the fate of T and B cells. However, somatic mutations or altered expression of NOTCH family members is frequently observed in lymphoid cell malignancies indicating that the signaling pathway underlying the activity of these molecules may play a relevant role in the survival and proliferation of neoplastic cells. This Research Collection hosts three papers on different characteristics of NOTCH signaling pathway involvement in lymphoid malignancies. In addition, the Research Collection hosts a review that mainly focuses on the activity of the NOTCH2 signaling pathway in mature B cell malignancies.

In T-ALL, the presence of genetic alterations of NOTCH1 has been widely described. NOTCH1 can be involved in the t(7;9) translocation or undergo somatic variations. A prolongation of NOTCH1 intracellular (IC) cell function is observed in all cases. Activated NOTCH1-IC promotes tumorigenesis by multiple tumor-promoting pathways, including NF-kB (1). In the first paper of this issue, Wang et al. show that treatment of T-ALL cell lines with the MALT1 inhibitor MI-2 interferes with NOTCH1-mediated activation of NF-kB. MALT-1 is part of a complex with BCL10 and CARMA1 (defined as CBM complex). The expression of an activated form of CARMA1 is necessary to allow NOTCH1-mediated deregulation of NF-kB. In the CBM complex, MALT1 plays a fundamental role by inactivating the NF-kB inhibitors. The incubation of T-ALL cell lines with MI-2 limits cell proliferation and induces cell apoptosis. In addition, using a T-ALL xenograft model, an improvement in mice survival is observed. Indeed, using a molecule interfering with NOTCH1-mediated NF-kB activation may represent an interesting strategy for targeted therapy of lymphoid malignancies with activated NOTCH1, such as T-ALL.

In Chronic Lymphocytic Leukemia (CLL), NOTCH1 somatic mutations are frequently encountered. These mutations mainly involve the intracellular (IC) PEST domain, resulting in delayed degradation of activated NOTCH1. In addition, in CLL cells, it has been shown that activation of the NOTCH1 signaling pathway can be intermingled with that activated by CLL cell B cell receptor engagement (2). This appears important in determining leukemic cell survival and clonal evolution. It has been widely demonstrated that NOTCH1 mutations negatively impact the CLL disease course (3). However, increased NOTCH1 activity in CLL cells has been observed independently of NOTCH1 mutations (4). In this issue, Baldoni et al. used Western Blot analysis to evaluate the presence of NOTCH1 activation (defined as ICN1+) in a cohort of 163 CLL patients and correlate these findings with the clinical course. Their findings indicate that ICN1+ patients have a time to first treatment (TFT) superimposable with NOTCH1 mutated patients. In addition, when combining ICN1+ and NOTCH1 mutated cases, these appear to be prognostically independent of CLL IGHV mutational status. Although NOTCH mutations involve NOTCH1 primarily, the authors show that activation of NOTCH2 (ICN2+) and expression of its ligand (JAG1) can be observed in CLL cells. However, the prognostic impact of ICN2+ and JAG1+ in CLL cells is still unclear and this point should be addressed in a cohort with more patients.

The role of NOTCH2 expression in CLL response to the anti-BCL2 agent Venetoclax was investigated by Fiorcari et al. These authors restricted their study to CLL patients with trisomy 12 who show lower levels of Interferon Regulatory Factor 4 (IRF4). IRF4 has been characterized as capable of interfering with NOTCH2 and BCL2 expression. Thus, trisomy 12 CLL cases show higher levels of NOTCH2 and BCL2 and, consequently, higher resistance to Venetoclax-induced apoptosis *in vitro*. In addition, the authors show that by inhibiting NOTCH2 and MCL1 expression, a higher sensitivity to Venetoclax is observed in CLL cells. There is no clinical evidence yet of a worse clinical outcome in trisomy 12 CLL patients treated with Venetoclax; however, the possible association of NOTCH2 expression with the emergence of Venetoclax resistance has not been studied as yet.

The role of NOTCH2 in the pathogenesis of CLL, Splenic Marginal Zone Lymphoma and Nodal Marginal Zone Lymphoma was reviewed in the final article of this issue by Mesini et al. The review recapitulates most of the structural and signaling features of the NOTCH family proteins, together with the evidence that NOTCH2 regulates the development of B1 and marginal zone B cells. The review also presents and discusses the pattern of altered expression or somatic mutations that can be observed in CLL and Marginal Zone lymphomas. The functional consequences and the role of these mutations still have to be fully understood to design targeted therapeutic approaches with minimal on-target and off-tumor side effects.

Overall, this collection contains new information and concepts related to the NOTCH signaling pathway in lymphoid disorders that can provide further ideas to expand the knowledge in the field.

## Author contributions

The authors have made a substantial, direct, and intellectual contribution to the work and approved it for publication.
